# Serotonin regulates prostate growth through androgen receptor modulation

**DOI:** 10.1038/s41598-017-15832-5

**Published:** 2017-11-13

**Authors:** Emanuel Carvalho-Dias, Alice Miranda, Olga Martinho, Paulo Mota, Ângela Costa, Cristina Nogueira-Silva, Rute S. Moura, Natalia Alenina, Michael Bader, Riccardo Autorino, Estêvão Lima, Jorge Correia-Pinto

**Affiliations:** 1 Life and Health Sciences Research Institute (ICVS), School of Medicine, University of Minho, 4710-057 Braga, Portugal; 2 ICVS/3B’s - PT Government Associate Laboratory, 4710-057 Braga/Guimarães, Portugal; 3 Department of CUF Urology and Service of Urology - Hospital of Braga, Braga, Portugal; 4 Department of Obstetrics and Gynecology, Hospital de Braga, Braga, Portugal; 5 Max Delbrück Center for Molecular Medicine, Robert-Rössle-Str. 10, Berlin, 13125 Germany; 6 Department of Pediatric Surgery, Hospital de Braga, Braga, Portugal

## Abstract

Aging and testosterone almost inexorably cause benign prostatic hyperplasia (BPH) in Human males. However, etiology of BPH is largely unknown. Serotonin (5-HT) is produced by neuroendocrine prostatic cells and presents in high concentration in normal prostatic transition zone, but its function in prostate physiology is unknown. Previous evidence demonstrated that neuroendocrine cells and 5-HT are decreased in BPH compared to normal prostate. Here, we show that 5-HT is a strong negative regulator of prostate growth. *In vitro*, 5-HT inhibits rat prostate branching through down-regulation of androgen receptor (AR). This 5-HT’s inhibitory mechanism is also present in human cells of normal prostate and BPH, namely in cell lines expressing AR when treated with testosterone. In both models, 5-HT’s inhibitory mechanism was replicated by specific agonists of *5-Htr1a* and *5-Htr1b*. Since peripheral 5-HT production is specifically regulated by tryptophan hydroxylase 1(Tph1), we showed that *Tph1* knockout mice present higher prostate mass and up-regulation of AR when compared to wild-type, whereas 5-HT treatment restored the prostate weight and AR levels. As 5-HT is decreased in BPH, we present here evidence that links 5-HT depletion to BPH etiology through modulation of AR. Serotoninergic prostate pathway should be explored as a new therapeutic target for BPH.

## Introduction

Benign prostatic hyperplasia (BPH) is one of the main causes of non-neurogenic lower urinary tract symptoms (LUTS) in the aging male^1,2^. The underlying mechanism responsible for BPH is not understood, and only elucidating the etiology of BPH will increase our ability to treat or even prevent its development.

Currently the most accepted hypothesis for the etiology of BPH is, that proposed by McNeal, in which BPH results from the reawakening of inductive potential in adult prostatic stroma in a specific prostatic region defined as transition zone^3–5^. This hypothesis claimed that the adult prostatic epithelium retains the ability to respond to inductive stromal signaling with new ductal branching morphogenesis^6,7^. However this hypothesis does not respond to the critical question of why this reawakening of human adult prostatic stroma occurs.

While there is no BPH without testosterone^8^, testosterone levels decrease with age^9,10^ and no direct correlation between testosterone concentration and prostate volume has been established yet^11^. Moreover, it is widely accepted that physiologic concentrations of testosterone provide an excess of testosterone for optimal prostatic growth suggesting that testosterone is not the etiologic factor responsible for BPH^12^. On the other hand, several reports have documented an up-regulation of the androgen receptor (AR) in BPH tissue, unveiling a potential role for AR in BPH etiopathogenesis^13–15^.

The neuroendocrine prostatic cells secrete various neuroendocrine factors with 5-HT being one of the most abundant. The peculiar morphology of some neuroendocrine cells with dendritic processes extending to lumen and projections surrounding the epithelial-stroma interface justify the hypothesis that neuroendocrine products, namely 5-HT, could regulate prostate growth^16^. Notably, neuroendocrine prostatic cells are mainly located in the transition zone of the normal human prostate^17^, where BPH originates^4^. However, comparing BPH tissue with normal transition zone (without BPH) the number of neuroendocrine cells is extraordinarily decreased^18–20^. Also 5-HT was shown to be significantly depleted in BPH tissue^19^. Furthermore, a recent study in a large cohort of Scandinavian men revealed that LUTS are associated with benign prostate enlargement and to decreased plasmatic 5-HT concentration^21^. These findings suggest a potential link between prostatic 5-HT depletion and BPH etiology; however, the function of 5-HT in regulation of benign prostate growth has never been studied.

We hypothesized that 5-HT had an inhibitory function over benign prostate growth and that suppression of prostatic 5-HT production could be responsible for benign prostatic growth. The aim of this study was to define the role of 5-HT in the regulation of benign prostatic growth and to test the pharmacologic modulation of the prostatic serotoninergic system as a new pharmacological target for BPH.

## Results

### 5-HT, 5-Htr1a, and 5-Htr1b specific agonists inhibits rat ventral prostate branching through AR down-regulation

**Figure 1:**
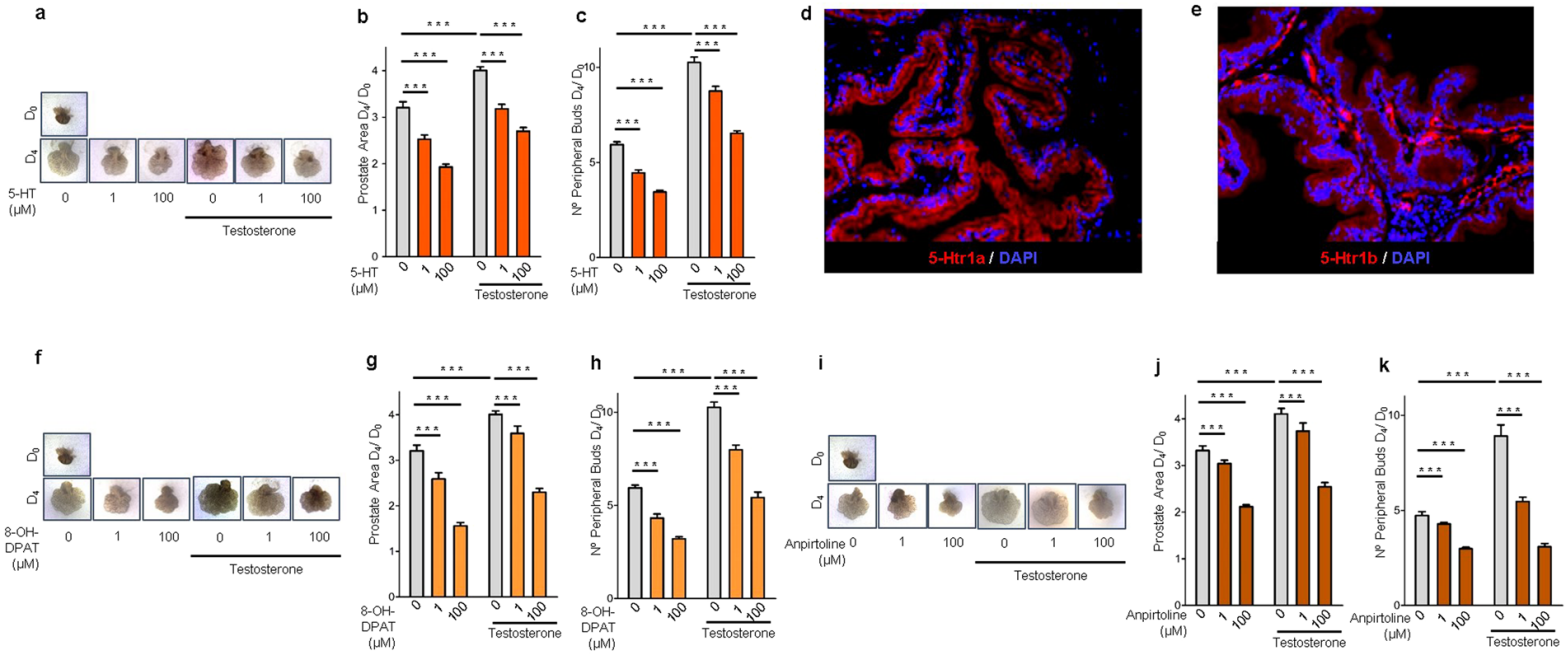
5-HT, *5-Htr1a* specific agonist and *5-Htr1b* specific agonist inhibit prostate branching morphogenesis. (**a**) Photographs of representative VPs at D_0_ and at D_4_ of culture treated with different 5-HT concentrations. (**b**) Morphometric analysis of the effect of 5-HT on VPs area and (**c**) number of peripheral buds (*n* ≥ 12 VPs per group). (**d**) Immunofluorescence analysis of *5-Htr1a* and (**e**) *5-Htr1b* expression in the rat prostate. (**f**) Photographs of representative VPs at D0 and at D4 of culture treated with different 8-OH-DPAT concentrations. (**g**) Morphometric analysis of the effect of 8-OH-DPAT on VPs area and (**h**) number of peripheral buds (*n* ≥ 12 VPs per group). (**i**) Photographs of representative VPs at D0 and at D4 of culture treated with different anpirtoline concentrations. (**j**) Morphometric analysis of the effect of Anpirtoline on VPs area and (**k**) number of peripheral buds (*n* ≥ 12 VPs per group). Error bars indicate s.e.m. ***p < 0.001; two-way ANOVA and Bonferroni *post hoc* test. VPs, ventral prostate explants; D_0_, day 0; D_4_, day 4; 5-HT, serotonin.

The new epithelial gland formation observed in BPH is normally seen only during prostate branching morphogenesis^22^. For this reason, we first tested the hypothesis that 5-HT could regulate prostate growth using *in vitro* cultures of rat ventral prostate explants (VPs) from P1 newborns. During 4 days in culture, 5-HT supplementation induced a significant dose-dependent inhibition of rat VPs growth (Fig. 1a), as expressed by decreased area (Fig. 1b), as well the number of peripheral explant buds (Fig. 1c). In medium conditions without additional testosterone supplementation, inhibitory effect of 5-HT over VPs growth was maximal at 100 µM where a reduction of 40% in prostate area D_4_/D_0_ (p < 0.001) and a reduction of 42% in the number of peripheral buds D4/D0 (p < 0.001) was observed in comparison to the control group (0 µM 5-HT). As expected, testosterone supplementation of VPs exerted a strong stimulatory effect on prostate branching morphogenesis, mainly in the number of peripheral buds (Fig. 1c), but again, 5-HT at 100 µM reduced 33% the prostate area D_4_/D_0_ (p < 0.001) and 36% the number of peripheral buds D4/D0 (p < 0.001) in comparison to control group (0 µM 5-HT + testosterone).

From all 5-HT receptors, *5-Htr1a* and *5-Htr1b* were the most extensively studied in the regulation of malignant prostate growth^23,24^, so we tested if these receptors could contribute to the 5-HT inhibitory function in normal prostate growth. By immunofluorescence, we found that both receptors are strongly expressed in rat prostate but with a slightly different distribution pattern with *5-Htr1a* predominantly expressed in prostate epithelium (Fig. 1d), while *5-Htr1b* being expressed both in epithelium and stroma (Fig. 1e). To determine the contribution of both receptors in 5-HT inhibition of prostate branching morphogenesis, VPs were treated with drugs that specifically activate *5-Htr1a* or *5-Htr1b*. The selective *5-Htr1a* agonist 8-OH-DPAT, (Fig. 1f,g and h) and the selective *5-Htr1b* agonist, anpirtoline, (Fig. 1i, j and k) induced a significant dose-dependent inhibition of VPs growth. The inhibitory effect was maximal in VPs supplemented with testosterone and treated with 100 µM of anpirtoline, where a reduction of 39% in prostate area D_4_/D_0_ (p < 0.001) and a reduction of 66% in the number of peripheral buds D4/D0 (p < 0.001) was observed in comparison to the control group (0 µM anpirtoline + testosterone).

**Figure 2:**
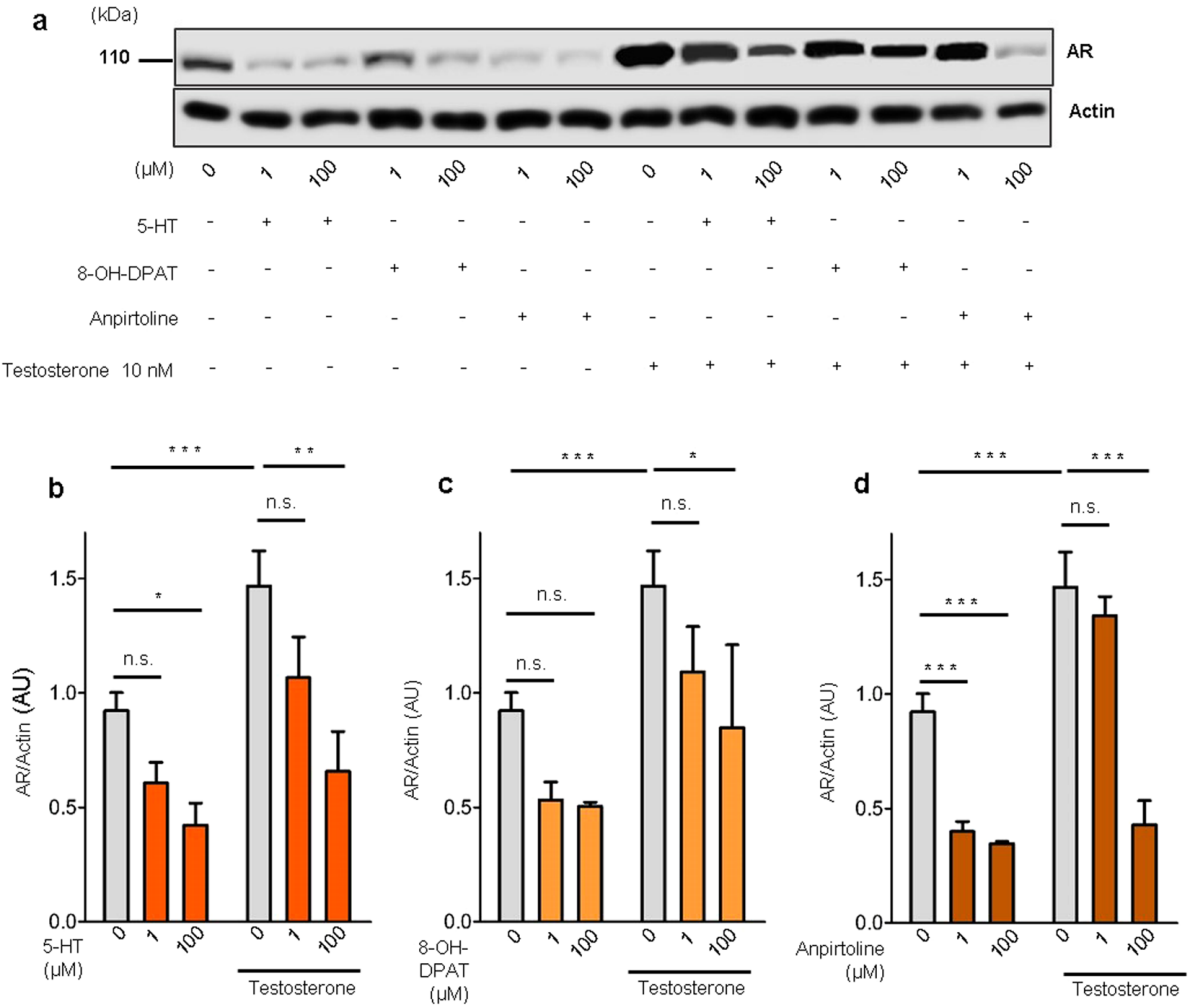
5-HT, *5-Htr1a* specific agonist and *5-Htr1b* specific agonist down-regulates AR expression in rat ventral prostate. (**a**) Western blot analysis of AR expression in prostate explants treated with increasing doses of 5-HT, specific 5-Htr1a agonist, 8-OH-DPAT, and specific 5-Htr1b agonist, anpirtoline. (**b**) Quantification of AR protein in VPs treated with different concentrations of 5-HT in medium conditions without or with testosterone supplementation, *n* ≥ 3 (each sample contained a pool of 4 VPs). (**c**) Quantification of AR protein in VPs treated with different concentrations of 8-OH-DPAT in medium conditions without or with testosterone supplementation, *n* ≥ 3 (each sample contained a pool of 4 VPs). (**d**) Quantification of AR protein in VPs treated with different concentrations of Anpirtoline in medium conditions without or with testosterone supplementation, *n* ≥ 3 (each sample contained a pool of 4 VPs). Error bars indicate s.e.m. *n.s*. non-significant; *p < 0.05; **p < 0.01; ***p < 0.001; two-way ANOVA and Bonferroni *post hoc* test. VPs, ventral prostate explants; AR, androgen receptor; AU, arbitrary units; 5-HT, serotonin.

Since, androgens are a major prostatic stimulatory factor, we asked if the 5-HT inhibitory effect was related to the AR stimulatory pathway. By western blot analysis we showed that testosterone supplementation induced AR up-regulation, but 5-HT treatment significantly decreased AR expression either with or without testosterone supplementation (Fig. 2a and b) suggesting that the inhibitory function of 5-HT could be related to inhibition of the AR pathway. Similarly, both the selective *5-Htr1a* agonist 8-OH-DPAT, (Fig. 2a and c) and the selective *5-Htr1b* agonist, anpirtoline, (Fig. 2a and d) induced a significant AR down-regulation, more evident in anpirtoline treated VPs. Taken together these results indicate that *in vitro* 5-HT inhibits rat prostate growth through *5-Htr1a* and *5-Htr1b*, by down-regulating AR.

### 5-HT, 5-Htr1a or 5-Htr1b specific agonists inhibit growth of androgen sensitive human benign prostate cells through AR down-regulation

**Figure 3:**
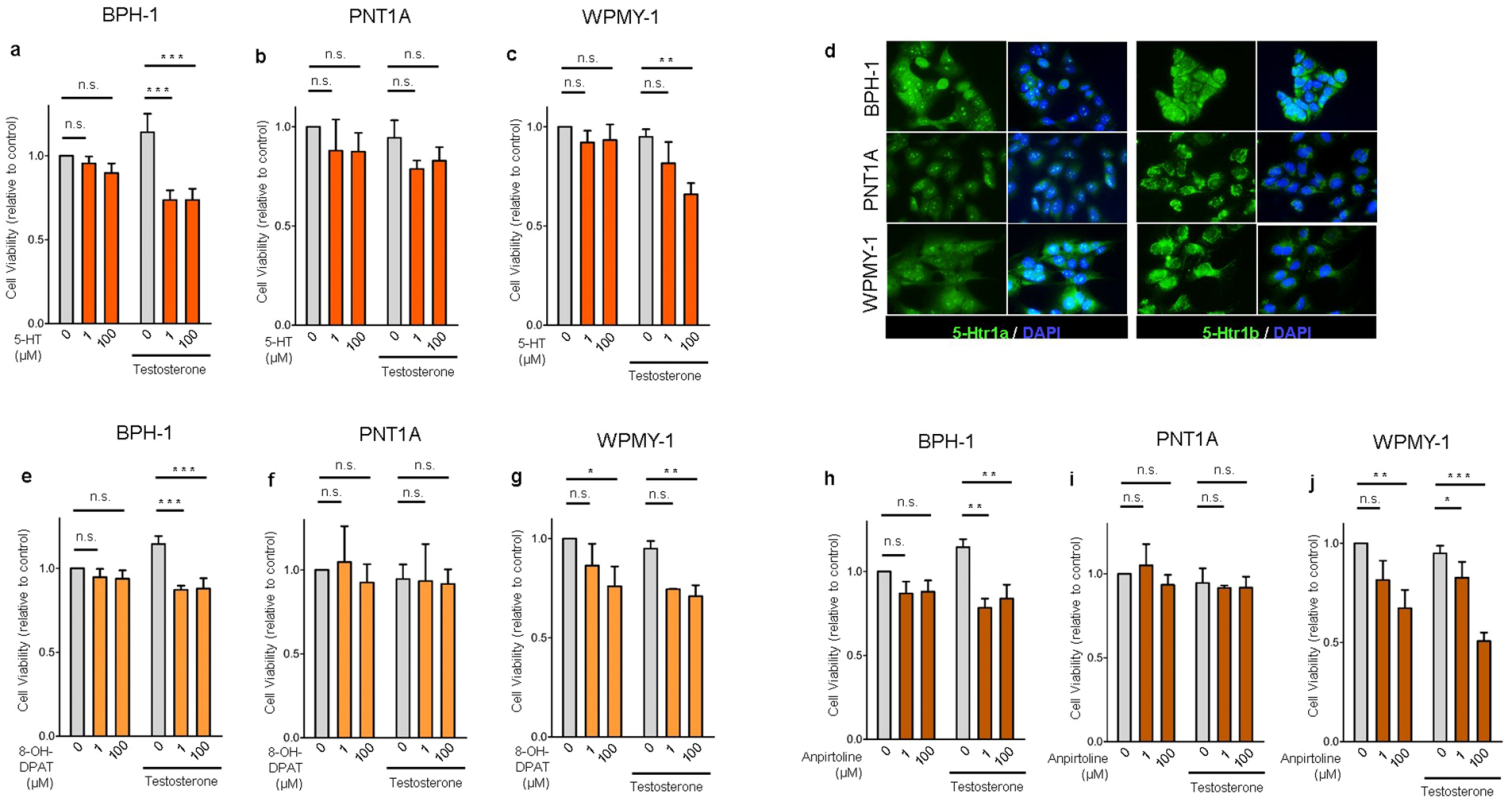
5-HT, *5-Htr1a* specific agonist and *5-Htr1b* specific agonist inhibits cell viability in BPH-1 and WPMY-1 human prostatic cells without any effect in PNT1A cells. (**a**,**b**,**c**) Effect of 5-HT on cell viability analyzed by MTS assay in BPH-1, PNT1A and WPMY-1 cells. (**d**) Immunofluorescence analysis of *5-Htr1a* and *5-Htr1b* expression in BPH-1, PNT1A and WPMY-1 cells. (**e**,**f**,**g**) Effect of *5-Htr1a* specific agonist, 8-OH-DPAT, and (**h**,**i**,**j**) *5-Htr1b* specific agonist, Anpirtoline, on cell viability analyzed by MTS assay in BPH-1, PNT1A and WPMY-1 cells. The data are expressed relative to control condition (0 µM 5-HT without testosterone supplementation) and were reproduced in at least three independent experiments. Error bars indicate s.e.m. *n.s*. non-significant; 5-HT, serotonin; *p < 0.05; **p < 0.01; ***p < 0.001; two-way ANOVA and Bonferroni post hoc test.

Next, we asked if this mechanism is also present in human prostate. With this purpose we performed *in vitro* 5-HT treatment of different human cell lines from epithelium of BPH (BPH-1), normal prostate epithelium (PNT1A) and normal prostate stroma (WPMY-1). We found that 5-HT significantly reduced cell viability of BPH-1 and WPMY-1 namely in the presence of testosterone but without changing PNT1A cell viability (Fig. 3a,b,c and Supplementary Fig. 1). The inhibitory effect of 5-HT was maximal in BPH-1 cells supplemented with testosterone. Under these conditions, 100 µM of 5-HT decreased cell viability by 35% compared to control (0 µM 5-HT + testosterone) (p < 0.001).

Next, we tested if the growth inhibitory function of 5-HT in androgen human prostate cells BPH-1 and WPMY-1 was mediated by *5-Htr1a* and *5-Htr1b*. First, we showed that both receptors are expressed in the three human prostate cell lines (Fig. 3d) and that their specific agonists significantly inhibited cell viability (Fig. 3e–j and Supplementary Fig. 1) almost exclusively in the presence of testosterone, but again only in BPH-1 and WPMY-1, without any inhibitory effect in PNT1A cells.

**Figure 4:**
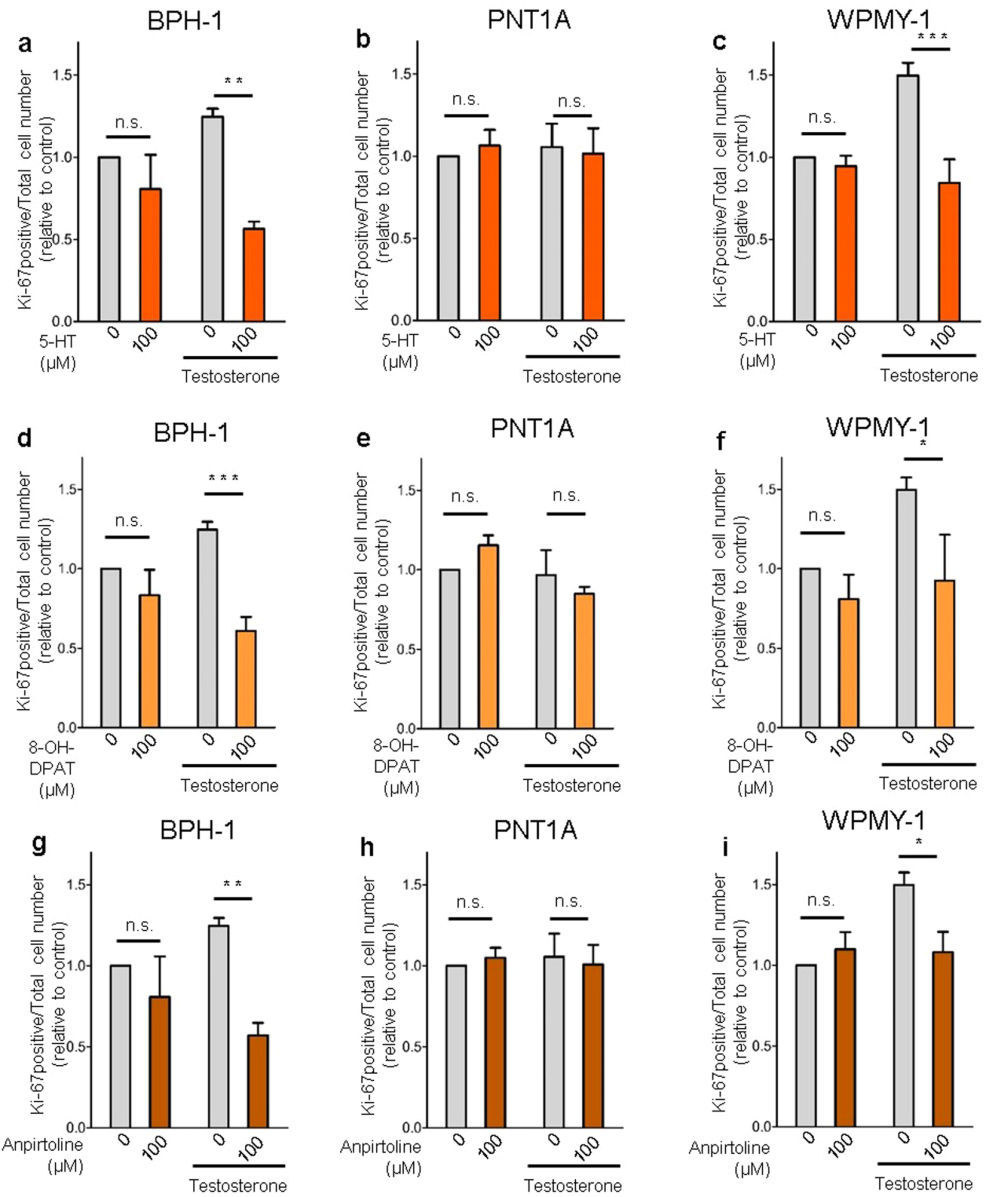
5-HT, *5-Htr1a* specific agonist and *5-Htr1b* specific agonist inhibits cell proliferation in BPH-1 and WPMY-1 human prostatic cells without any effect in PNT1A cells. (**a**,**b**,**c**) Effect of 5-HT, (**d**,**e**,**f**) *5-Htr1a* specific agonist, 8-OH-DPAT, and (**g**,**h**,**i**) *5-Htr1b* specific agonist, Anpirtoline, on cell proliferation of BPH-1, PNT1A and WPMY-1 cells as quantified by Ki-67 positive cells/total cell number ratio. The data are expressed relative to control condition (0 µM 5-HT without testosterone supplementation) and were reproduced in at least three independent experiments. Error bars indicate s.e.m. *n.s*. non-significant; 5-HT, serotonin; *p < 0.05; **p < 0.01; ***p < 0.001; two-way ANOVA and Bonferroni post hoc test.

Additionally, Ki-67 staining confirmed that proliferation of BPH-1 and WPMY-1 cells supplemented with testosterone was significantly reduced by 5-HT treatment (Fig. 4a and c), while PNT1A cells proliferation was not affected (Fig. 4b). Similarly, both specific agonists of *5-Htr1a* and *5-Htr1b* strongly inhibited cell proliferation (Fig. 4d–i) but again only in BPH-1 and WPMY-1 cells.

**Figure 5:**
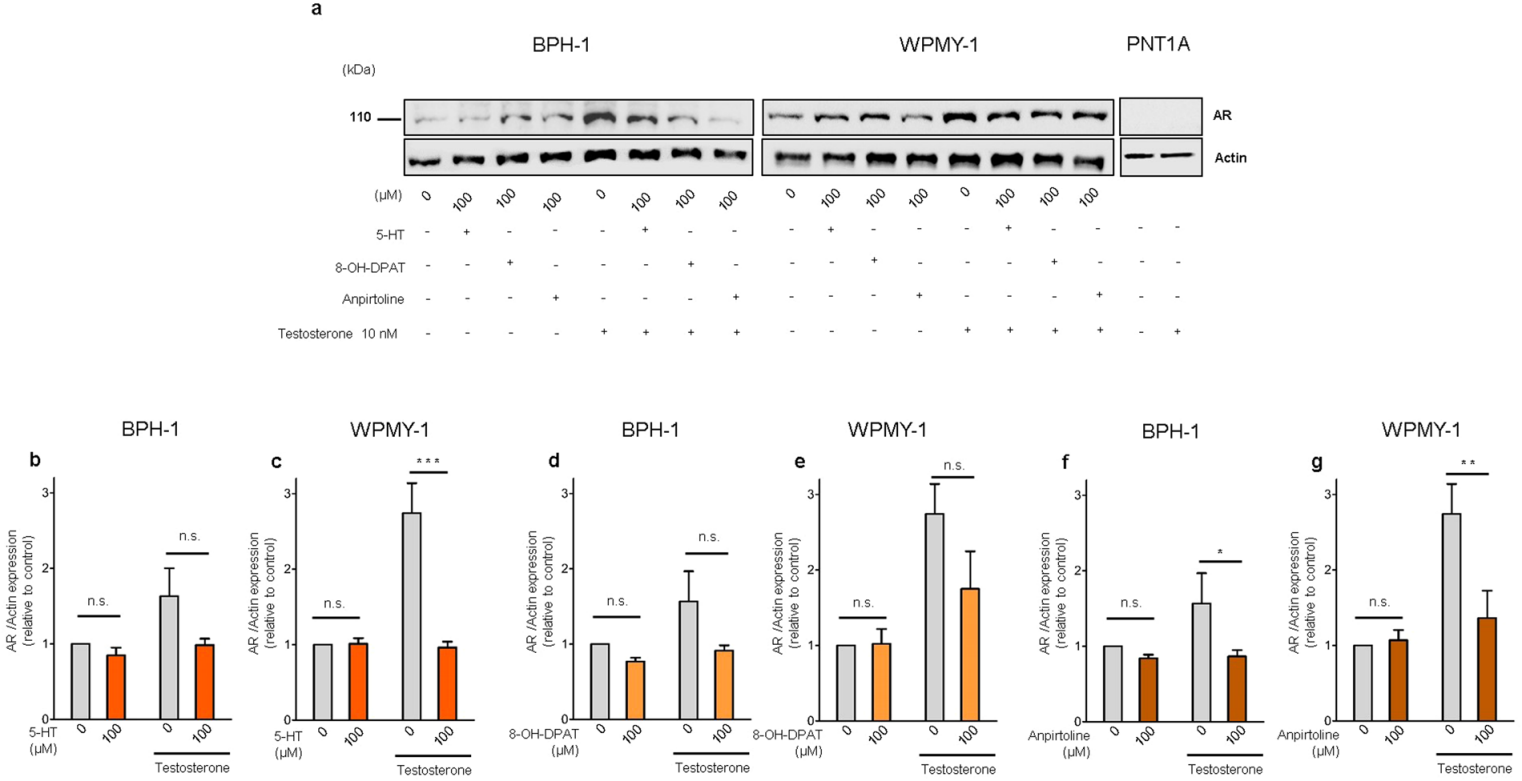
5-HT, *5-Htr1a* specific agonist and *5-Htr1b* specific agonist down-regulates AR expression in human prostatic cells. (**a**) Western blot analysis of AR expression in the three cell lines after 5-HT, 8-OH-DPAT and anpirtoline treatment. (**b**) Quantification of AR in BPH-1 and (**c**) WPMY-1 cells after 5-HT treatment in medium conditions without or with Testosterone supplementation. (**d**) Quantification of AR protein levels in BPH-1 and (**e**) WPMY-1 cells after 8-OH-DPAT treatment in medium conditions without or with Testosterone supplementation. (**f**) Quantification of AR protein levels in BPH-1 and (**g**) WPMY-1 cells after Anpirtoline treatment in medium conditions without or with Testosterone supplementation. The data are expressed relative to control condition (0 µM 5-HT without testosterone supplementation) and were reproduced in at least three independent experiments. Full, uncropped gel images are shown. Error bars indicate s.e.m. *n.s*. non-significant; *P < 0.05; **P < 0.01; ***P < 0.001; two-way ANOVA and Bonferroni *post hoc* test. AR, androgen receptor; 5-HT, serotonin.

Next, we investigated if this inhibitory function of 5-HT, *5-Htr1a* and *5-Htr1b* specific agonists was related to changes in the AR pathway. We observed that testosterone induced an up-regulation of AR in both BPH-1 and WPMY-1 cells. 5-HT, *5-Htr1a* and *5-Htr1b* specific agonists inhibited the AR up-regulation induced by testosterone in both BPH-1 and WPMY-1 cells (Fig. 5b). Regarding 5-HT effect in AR down-regulation this was very significant in WPMY-1 cells after testosterone supplementation (p < 0,001) while in BPH-1 cells a non-significant down-regulation of AR was observed (Fig. 5c and d). Also for both *5-Htr1a* and *5-Htr1b* specific agonists only Anpirtoline induced a significant down-regulation of AR in both BPH-1 and WPMY-1 cells (Fig. 5d,e,g,h). Additionally, by immunofluorescence analysis we observed that expression of AR after testosterone treatment was decreased in BPH-1 cells treated with 5-HT, *5-Htr1a* and *5-Htr1b* specific agonists (Supplementary Fig. 2).

Remarkably, the absence of inhibitory action of 5-HT, *5-Htr1a* and *5-Htr1b* specific agonists on PNT1A cells viability and proliferation, even in presence of testosterone, co-existed with a complete absence of AR expression in these cells (Fig. 5a). These data strongly argue that 5-HT’s inhibitory function on growth of benign human prostate cells is related with the suppression of the AR pathway.

### *In vivo* ablation of peripheral 5-HT synthesis in mice induces benign prostatic growth

**Figure 6:**
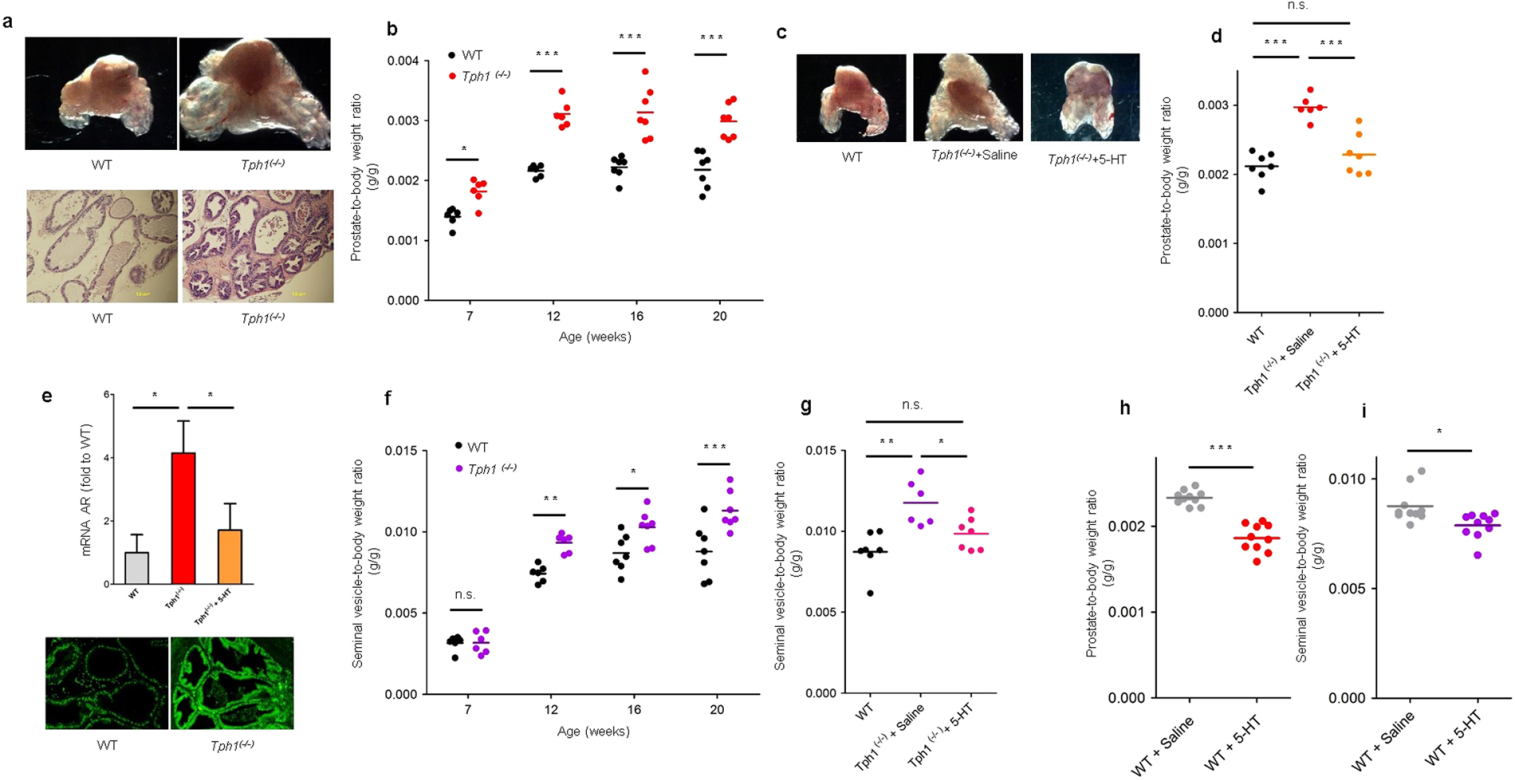
Genetic deletion of Tph1 increases prostate gland mass. (**a**) Representative photographs of prostates from 20 week-old wild-type and *Tph1* ^−/−^ mice (Top). Images from H&E staining of wild-type and *Tph1* ^−/−^ prostates (*n* = 5 per genotype) (Bottom). (**b**) Prostate-to-body weight ratio of *Tph1* ^−/−^ mice compared to wild-type at different ages (*n* = 6–7 for wild-type and *Tph1* ^−/−^ mice, for each time point). (**c**) Representative photographs of prostates and (**d**) prostate-to-body weight ratio from 20 week-old wild-type, *Tph1* ^−/−^ treated with saline and *Tph1* ^−/−^ treated with 5-HT (*n* = 7 WT; *n* = 6 *Tph1* ^−/−^ +Saline; *n* = 7 *Tph1* ^−/−^ + 5-HT). (**e**) qRT-PCR for AR expression in dorsolateral lobe of 20 week-old wild-type, *Tph1* ^−/−^ treated with saline and *Tph1* ^−/−^ treated with 5-HT (*n* = 4 wild-type; *n* = 4 *Tph1* ^−/−^ +Saline; *n* = 3 *Tph1* ^−/−^ + 5-HT) (Top). Immunofluorescence analysis of AR expression in dorsolateral prostate of WT and *Tph1* ^−/−^ mice (200x) (Bottom). (**f**) Seminal vesicle-to-body weight ratio of *Tph1* ^−/−^ mice compared to wild-type mice at different ages (n = 6–7 for wild-type and *Tph1* ^−/−^; mice, for each time point). (**g**) Seminal vesicle-to-body weight ratio from 20 week-old wild-type, *Tph1* ^−/−^ treated with saline and *Tph1* ^−/−^ treated with 5-HT (*n* = 7 WT; *n* = 6 *Tph1* ^−/−^ + Saline; *n* = 7 *Tph1* ^−/−^ + 5-HT). (**h**) prostate-to-body weight ratio and (**i**) seminal vesical-to-body weight ratio in wild-type mice treated with Saline compared to wild-type mice treated with 5-HT during 10 consecutive days (n = 10 for each group). *n.s*. non-significant; AR, androgen receptor; 5-HT, serotonin; *p < 0.05; **p < 0.01; ***p < 0.001; (B,F) two-way and (D,G) one-way ANOVA and Bonferroni *post hoc* test. (H,I) Student *t* test.

5-HT synthesis is initiated by tryptophan hydroxylase (Tph). Tph type 1 (Tph1) and 2 (Tph2) regulate 5-HT production in non-neuronal and neuronal tissues, respectively^25,26^. The majority of 5-HT in the body is produced by Tph1. In fact, *Tph1* ^−/−^ mice exhibit very low levels of circulating 5-HT, while brain serotonin is not affected^25^. Based on our *in vitro* findings which suggest that 5-HT has a strong inhibitory action on prostate growth through down-regulation of AR, we used *Tph1* ^−/−^ mice to evaluate the effect of peripheral 5-HT depletion on mouse prostate gland growth. Remarkably, *Tph1* ^−/−^ mice exhibited a significantly 37% higher prostate-to-body weight ratio compared to wild-type at 20 weeks (p < 0.001) (Fig. 6a and b) without changes in body weight (Supplementary Fig. 3a). Interestingly, histology of the prostate gland revealed that *Tph1* ^−/−^ mice exhibit areas of hyperplasia in epithelium and stroma (Fig. 6a, lower panel). To determine if 5-HT treatment could revert higher prostate mass in *Tph1* ^−/−^ mice, we performed intraperitoneal injections of 5-HT at 10 consecutive days. 5-HT treatment resulted in significant mass reduction in prostate gland compared to levels similar to the wild-type (Fig. 6c and d) again without affecting animal weight (Supplementary Fig. 3b). Next, we asked if the higher prostate mass in *Tph1* ^−/−^ mice was associated with different expression of AR. We could not demonstrate significant differences of AR expression by western blot analysis in total prostate (data not shown), however by immunofluorescence dorsolateral prostate of *Tph1* ^−/−^ mice appeared to express more AR (Fig. 6e, lower panel). So, we investigated and demonstrated by qRT-PCR that the dorsolateral prostate of *Tph1* ^−/−^ mice has increased levels of AR mRNA expression, while 5-HT treatment partially restores it to levels to wild-type mice (Fig. 6e, upper panel), reinforcing our hypothesis.

It is well established that castration induces a strong reduction in size of the prostate gland as well as in seminal vesicles, while testosterone supplementation makes both organs return to normal size^27,28^. Interestingly, also seminal vesicles of *Tph1* ^−/−^ mice were significantly larger than the ones of wild-type (Fig. 6f) suggesting that 5-HT could regulate androgen sensitivity not only in prostate gland but also in seminal vesicles. Again, we observed that the mass of seminal vesicles was partially restored by 5-HT treatment in *Tph1* ^−/−^ mice (Fig. 6g).

Lastly, we tested if wild-type mice challenged with 5-HT treatment continue responding to 5-HT’s inhibitory action, and we demonstrated that both prostate gland (Fig. 6h) as well as seminal vesicle mass (Fig. 6i) were reduced while animal weight was not affected (Supplementary Fig. 3c).

## Discussion

Currently, the etiology of BPH is unknown. However, it is accepted today that BPH is a consequence of aging and the simultaneous presence of testosterone^3^. BPH almost universally affects human males and a significant part of men will develop bothersome LUTS because of benign prostate enlargement. Although some of these men respond to current medical treatment (mainly α_1_-adrenoreceptors antagonists and 5 α-reductase inhibitors) a large portion continues to need a surgical procedure to treat resistant LUTS or have even more serious complications of BPH^29^, creating the emerging necessity for novel therapies.

In this study, we investigated the function of 5-HT in the regulation of non-malignant prostatic growth. Here, we demonstrated for the first time in several *in vitro* and *in vivo* models that 5-HT is a powerful negative regulator of prostatic growth through down-regulation of AR. We found that *5-Htr1a* and *5-Htr1b* are strongly expressed in the rodent prostate gland as well in human benign prostate cells, and that both receptors could mediate the inhibitory action of 5-HT on prostate growth.

**Figure 7:**
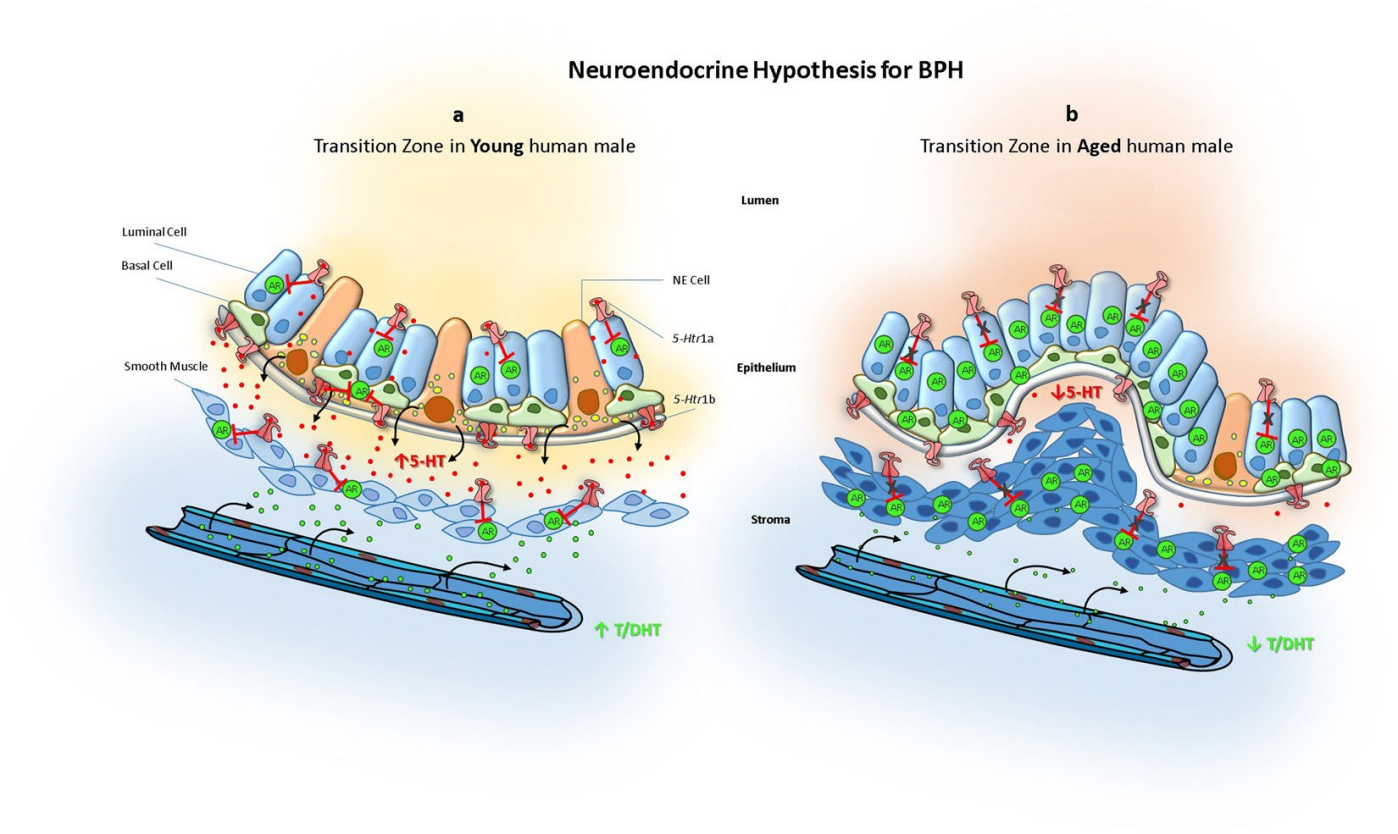
Neuroendocrine hypothesis for etiopathogenesis of benign prostatic hyperplasia. (**a**) In young human male, prostate transition zone is enriched with 5-HT producing neuroendocrine cells. Serotonin is secreted to the epithelium-stroma interface and through activation of *5-Htr1a* and *5-Htr1b*, both in epithelium and stroma, the expression of AR is decreased. Yet, more testosterone is delivery to prostate, down-regulated AR limits benign prostate growth. (**b**) In aged human male, transition zone loses 5-HT producing neuroendocrine cells causing a depletion in local 5-HT. As a consequence *5-Htr1a* and *5-Htr1b* release their inhibition over AR expression. Although with aging the delivery of testosterone to the prostate is decreased the up-regulation of AR induces the development of BPH. 5-HT, serotonin; AR, androgen receptor; DHT, Dihydrotestosterone; NE, neuroendocrine; T, testosterone.

Our *in vitro* and animal findings lead us to propose a new mechanism to explain the development of BPH in humans (Fig. 7). Our proposed model explains how the depletion of neuroendocrine cells and serotonin observed in prostatic transition zone with aging^18–20^, could be the etiologic factor responsible for the initiation and progression of BPH. In our model, the depletion of serotonin induces an up-regulation of androgen receptor in the prostatic transition zone leading to the stimulation of benign prostatic growth in this specific prostatic region.

Our neuroendocrine model for the etiopathogenesis of BPH would resolve an intriguing question about the crucial participation of androgens in the development of BPH. Previous studies have showed that testosterone does not increase with aging and some studies have even reported that plasmatic testosterone is decreased in the aging human male^9–11^. In a similar way, intraprostatic testosterone, in particular DHT, are not increased in BPH comparatively to normal prostate^30^ and even the administration of testosterone in supra-physiologic concentrations to eugonadal men does not induce the development of BPH^31,32^. This data were the basis for the saturation model of prostate growth proposed by Morgentaler *et al*. suggesting that prostate gland growth is extraordinarily sensitive to low androgen concentrations (near the castrate range) but insensitive to androgen concentrations above a certain saturation point^12^. The critical point, in saturation model is that the rate-limiting step for prostatic growth, which is the concentration of AR. Recent studies demonstrated that AR is in fact up-regulated in stroma and epithelium of BPH tissue comparatively to normal prostate, implicating AR in etiophatogenesis of BPH^13–15^. Our findings seem to provide an explanation for this current view about the participation of androgens in the development of BPH. In this way, the loss of neuroendocrine cells and serotonin in prostatic transition zone up-regulates the AR and then permits the development of BPH, even with a decreased plasmatic concentration of androgens observed in the aging male.

Our first *in vitro* experimental approach focused on the function of 5-HT in the regulation of rat prostate branching morphogenesis. Because BPH is the result of new branching morphogenesis, this model permits the study the influence of 5-HT exactly in the mechanism by which BPH develops and progresses. Here, we demonstrate that 5-HT strongly inhibit the branching morphogenesis of the prostate gland through down-regulation of AR. In fact, other organs like the prostate which have a development process of branching morphogenesis, such as the mammary gland, 5-HT also have demonstrated a development inhibitory action^33^.

Although new in the prostate gland, the serotoninergic inhibitory mechanism through down-regulation of AR, is well known in the brain. In fact, the complete masculinization of the brain is dependent of a perinatal surge of testosterone and a simultaneous decrease in hypothalamic 5-HT concentrations^34^. In the brain, it has been demonstrated that regulation of 5-HT concentration is crucial for normal sexual differentiation, where 5-HT down-regulates AR^35,36^. In agreement with our findings, Sayed *et al*. demonstrated that dapoxetine decreased AR expression and prevents testosterone-induced BPH in rats^37^. However, both in brain and prostate the full mechanism responsible for this down-regulation remains to be elucidated.

The most described 5-HT receptors in prostatic cells, are the *5-Htr1a* and *5-Htr1b*. Therefore, we characterize its expression in rat prostate for the first time. As we demonstrated both *5-Htr1a* and *5-Htr1b* are strongly expressed in rat prostate and the activation of these receptors resulted in a significant inhibition of prostate branching morphogenesis, which also occurred through down-regulation of AR. In human prostatic cells the function of 5-HT, *5-Htr1a*, and *5-Htr1b* have been previously studied but only in malignant cells^23,24^. These previous reports showed that 5-HT has a proliferative effect in several malignant cell lines through *5-Htr1a* and *5-Htr1b*. Curiously, this stimulatory effect was mainly evident in androgen-insensitive cells^23^. Here we studied, for the first time, the function of 5-HT, *5-Htr1a*, and *5-Htr1b* in human benign prostatic cells. We demonstrated that 5-HT inhibits proliferation of androgen sensitive benign prostatic cells, and this inhibitory function was associated to a down-regulation of AR. This different growth function of 5-HT in benign and malignant cells remains unexplained but the predominant stimulatory effect of 5-HT in androgen-insensitive malignant cells suggests that castration resistance could change the phenotypic response of prostatic cell to neuroendocrine products.

Finally, to test *in vivo* our mechanistic approach to the etiopathogenesis of BPH, we genetically ablated the peripheral production of 5-HT. Using Tph1^−/−^ mice we demonstrated that prostatic 5-HT depletion induces benign prostatic growth. The inhibition of peripheral 5-HT synthesis in mice, through genetic deletion of Tph1, simulates a decrease in the prostate transition zone 5-HT observed in the aging male. This led us to propose that the decrease in neuroendocrine cells and 5-HT in the human transition zone could contribute to the development of BPH. The increased mass of *Tph1* ^−/−^ prostates was associated to an up-regulation of AR in dorso-lateral prostate samples suggesting that, at least in part, the excessive prostatic growth in *Tph1* ^−/−^ could be attributed to AR up-regulation.

In conclusion, our findings suggest that 5-HT is a strong negative regulator of prostate growth through AR down-regulation. As 5-HT is decreased in BPH, we present here evidence that links 5-HT-producing neuroendocrine cell depletion to BPH etiology. Therefore, this new described serotoninergic inhibitory pathway over benign prostatic growth should be explored as a new target for BPH treatment.

## Methods

### Ethics and animal work

Mice and rats were maintained in accordance with the guidelines of “*Guide to the Care and Use of Experimental Animals”* National Academy of Science, and the EU Directive 2010/63/EU. This study was approved by the Animal Ethics Committee of the Institution were the study was performed (SECVS 003/2016) and by the National Competent Authority for Animal Protection (DGAV 0421/000/000/2016).

### Drugs

5-HT and testosterone were purchased from Sigma-Aldrich (St Louis, Missouri). The *5-Htr1a* specific agonist, 8-OH-DPAT and the *5-Htr1b* specific agonist, Anpirtoline, were purchased from Tocris-Bioscience (Bristol, UK).

### Rat ventral prostate cultures

Newborn male *Sprague-Dawley* rats were sacrificed 24-hours after birth. Ventral prostate lobes were microdissected using a stereomicroscope (Leica MZ6, Switzerland) and processed for organ culture. Organ culture was performed as previously described^38^. Briefly, rat ventral prostates (VPs) from P1 newborns were cultured for 4 days at 37 °C in a humidified atmosphere of 5% CO_2_. Medium and VPs were transferred to porous membranes (Millicell CM filters, Millipore Corp., Bedford, Massachusetts) in 12 well plate for floating explant cultures. Each VPs were dipped into 500 μl of 1:1 mixture of DMEM and Ham’s F-12 nutrient supplemented with 100 μg/mL streptomycin, 100 units/mL penicillin, 10 µg/mL transferrin and 10 µg/mL insulin. Media were replenished at 48 hours of culture. Branching morphogenesis in all groups was monitored daily by a stereomicroscope and photographs were taken at day 0 and day 4. The number of peripheral buds was manually counted and the prostate tissue area was measured in ImageJ using the beProstate plugin (Version 1.0) (developed by Biomedical Engineering Solutions Research Group, Life and Health Sciences Research Institute, University of Minho; available at http://www.besurg.com/sites/default/files/beProstateApp.zip). The differences between day 0 (D0: 0 hours) and day 4 (D4: 96 hours) of culture, were expressed as D4/D0 ratio. A total of 427 VPs were cultured divided in three experimental groups: 5-HT, 8-OH-DPAT and anpirtoline. For each experimental group a dose-effect approach was used. Furthermore, each experimental group was cultured either with or without testosterone supplementation of media ([testosterone] = 10^−8^ M).

### Human prostate cell lines cultures

Three human cell lines were used: PNT1A and WPMY-1 were obtained from American Type Culture Collection (ATCC, Manassas, Virginia) and BPH-1 which was obtained from DSMZ (German Collection of Microorganisms and Cell Cultures, Braunschweig, Germany). All the cell lines were maintained in Dulbecco’s modified Eagle’s medium (DMEM 1x, high glucose; Gibco, Invitrogen, Grand Island, New York) supplemented with 10% FBS (Gibco, Invitrogen, Grand Island, New York) and 1% penicillin/streptomycin solution (DMEM-10), at 37 °C and 5% CO_2_. For the viability assay, the cells were plated into 96-well plates at a density of 3 × 10^3^ cells per well and allowed to adhere overnight in DMEM medium containing 10% FBS. Subsequently, the cells were treated with increasing concentrations of 5-HT, 8-OH-DPAT and Anpirtoline diluted in 0.5% FBS culture medium, with or without testosterone (10^−8^M) supplementation. After 72 hours of incubation, cell viability was quantified using CellTiter 96 Aqueous Cell Proliferation Assay (MTS) (Promega, Madison, Wisconsin). The mean percentage of viable cells relative to the vehicle alone (considered as 100% viability) was determined, and the final results were expressed in relation to the control (adjusted to 1). For the proliferation assay, we evaluated the number of Ki-67 positive cells by immunofluorescence. The cells were plated in glass coverslips placed into 12-well plates at a density of 5 × 10^4^ cells per well, and allowed to adhere overnight. Subsequently, the cells were treated with 5-HT (100 µM), 8-OH-DPAT (100 µM) and Anpirtoline (100 µM) diluted in 0.5% FBS culture medium, with or without testosterone (10^−8^M) supplementation. The total number of cells and the Ki-67 positive cells were manual counted using a fluorescence microscopy (BX16; Olympus). The ratio of Ki-67 positive cells per total number of cells was determined, and the final results were expressed in relation to the control (adjusted to 1).

### Tph1^−/−^ Mice and *in vivo* studies

Only male mice were used for *in vivo* experiments. They were housed in specific pathogen-free conditions in a room maintained at a constant temperature of 23 °C on a 12-h light-dark cycle. Food and water were provided *ad libitum*. All treatment groups were age matched and randomized to treatment at the initiation of an experiment. The researchers performing the experiments were blinded to experimental groups during all testing. Animals were excluded from analysis if signs of fight with skin lesions were present. *Tph1* ^−/−^ mice on a C57BL/6 background were provided by M. Bader (Max Delbrück Center for Molecular Medicine, Berlin, Germany). For assessment of the morphological evolution with age both male wild-type and *Tph1* ^−/−^ mice were sacrificed at different time points (at least 6 animals for both groups at each time-point): 7, 12, 16 and 20 weeks-old. For pharmacological studies, wild-type and *Tph1* ^−/−^ mice with 19 week-old were treated daily with intraperitoneal injections of 0.9% saline or 5-HT (100 mg/Kg) during 10 consecutive days (at least 6 animals for each group). Mice were sacrificed and prostate tissue (all lobes combined) and seminal vesicles were micro dissected away from other urogenital and fat tissues. Total prostate was weighted immediately after dissection. The right lobes were separated from the left and processed for histology or western blotting. For histologic analysis hemi-right prostate was fixed in 10% PFA, processed and embedded in paraffin and stained with hematoxylin and eosin (H&E). Hemi-left prostate was separated in three lobes (ventral, dorsolateral and anterior) and processed to qRT-PCR.

### Immunofluorescence analysis

Immunofluorescence for AR, Ki-67, *5-Htr1a* and *5-Htr1b* was performed on formalin-fixed and paraffin-embedded rat ventral prostates or in human prostate cell lines. Briefly, deparaffinized and rehydrated slides were submitted to adequate heat-induced antigen retrieval for 20 min at 98 °C with 10 mM citrate buffer (pH 6.0). Regarding cell lines, all of them were plated in glass coverslips placed into 12-well plates at a density of 5 × 10^5^ cells per well, and allowed to adhere overnight. Then, the cells were fixed in cold methanol by 5 minutes at −20 °C. In both paraffin and cell, for block unspecific ligations the cells/tissues were incubated with a solution of PBS containing 10% FBS for 30 minutes at room temperature followed by incubation with a primary antibody against AR (1:1000 dilution; sc-816: Santa Cruz Biotechnology, Santa Cruz, California), Ki-67 (1:100 dilution, AP10244C; Gennova, Sevilla, Spain), *5-Htr1a* (1:100 dilution; sc-10801: Santa Cruz Biotechnology, Santa Cruz, California) and *5-Htr1b* (1:100 dilution; sc-28937: Santa Cruz Biotechnology, Santa Cruz, California). The cells/tissues were then washed in a PBS solution with 0.5% FBS and incubated with a goat anti-rabbit antibody conjugated with FITC for cells and with TRITC for tissues (dilution 1:500, Life Technology, Carlsbad, California) for 1 hour at room temperature in the dark. Finally, the cells were counterstained with 40,6-diamidino-2-phenylindole (DAPI). The images were obtained using a fluorescence microscopy (BX16; Olympus).

### Western blot analysis

Western blot analysis for androgen receptor (AR) was done in both VPs, mouse prostate tissue and in human cell lines. All the samples were properly processed for western blot analysis and lysed in a buffer containing 50 mM Tris pH 7.6–8, 150 mM NaCl, 5 mM EDTA, 1 mM Na3VO_4_, 10 mM NaF, 10 mM NaPyrophosphate, 1% NP-40 and 1/7 of Protease cocktail inhibitors (Roche). Western blotting was done using standard 10% SDS-PAGE gels, loading 20 µg of protein per lane. For AR detection a specific antibody was used (1:1000 dilution; sc-816: Santa Cruz Biotechnology, Santa Cruz, California). β-Actin was used for loading control (1:500 dilution; sc-1616; Santa Cruz Biotechnology, Santa Cruz, California). After incubation with appropriate secondary antibodies, they were detected by chemiluminescence (Thermo Scientific Pierce ECL Western Blotting) in ChemiDoc™ XRS + System (Bio-Rad). Quantification of western blot results was done using the band densitometry analysis, performed with ImageJ software.

### RNA extraction and qRT-PCR

Total RNA was isolated from the dorsolateral prostate of different groups with Trizol (Invitrogen, Carlsbad, California). Then, after quantification using the NanoDrop^®^, 500 ng of total RNA was reverse transcribed into first strand cDNA using the iScript™ cDNA Synthesis Kit (Bio-Rad Laboratories, Hercules, California). Primers used to measure the expression levels of AR was designed using the Primer3 software, on the basis of the respective GenBank sequence. All accession numbers and primer sequences are available on request. The reference gene for hypoxanthine guanine phosphoribosyl transferase (*Hprt*) (accession number from GenBank: NM_013556) was used as an internal standard for the normalization of the expression of selected transcripts. qRT-PCR was performed on a CFX 96TM real time system instrument (Bio-Rad Laboratories, Hercules, California), with the QuantiTect SYBR Green RT-PCR reagent kit (Qiagen, Hamburg, Germany), using equal amounts of RNA from each one of the samples. Product fluorescence was detected at the end of the elongation cycle. All melting curves exhibited a single sharp peak at the expected temperature.

### Statistics

Data are presented as mean ± SEM. Statistical analysis was performed using GraphPad Prism by Student’s *t* test or ANOVA where appropriate. A Bonferroni *post hoc* test was used to test for significant differences revealed by ANOVA. Statistical significance was confirmed at p < 0.05.

## Electronic supplementary material

Supplementary Information
