# Supplementary material for: Serotonin regulates prostate growth through androgen receptor modulation
Source: Sci Rep. 2017 Nov 13;7:15428. doi: 10.1038/s41598-017-15832-5 (PMC5684231; doi:10.1038/s41598-017-15832-5)
Supplement: Supplementary file 1 — Supplementary Information [file 41598_2017_15832_MOESM1_ESM.pdf]

Serotonin regulates prostate growth through androgen receptor modulation

Emanuel Carvalho-Dias[1,2,3*], Alice Miranda[1,2], Olga Martinho[1,2], Paulo Mota[1,2,3], Ângela Costa[1,2], Cristina Nogueira-Silva[1,2,4], Rute S Moura[1,2], Natalia Alenina[5], Michael Bader[5], Riccardo Autorino[1,2], Estêvão Lima[1,2,3], Jorge Correia-Pinto[1,2,6].

Supplementary Figure 1

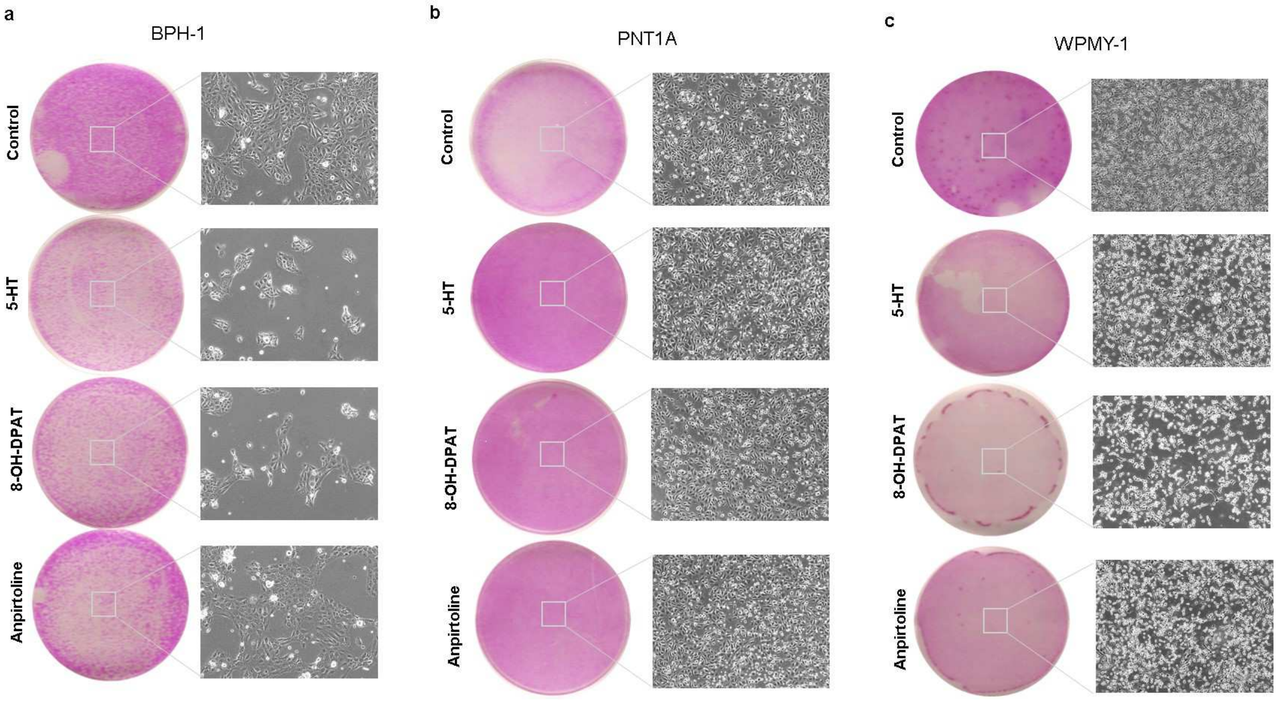

Supplementary Figure 1. Effect of 5-HT, *5-Htr1a* specific agonist and *5-Htr1b* specific agonist on cell viability and morphology of human prostatic cells treated with testosterone. Effect of 5-HT, *5-Htr1a* specific agonist and *5-Htr1b* specific agonist on cell viability and morphology after 72 hours of treatment in BPH-1 **(a)**, PNT1A **(b)** and WPMY-1 cells **(c)**. Representative example of three independent experiments, 1 field of Microscope, 400X.

Supplementary Figure 2

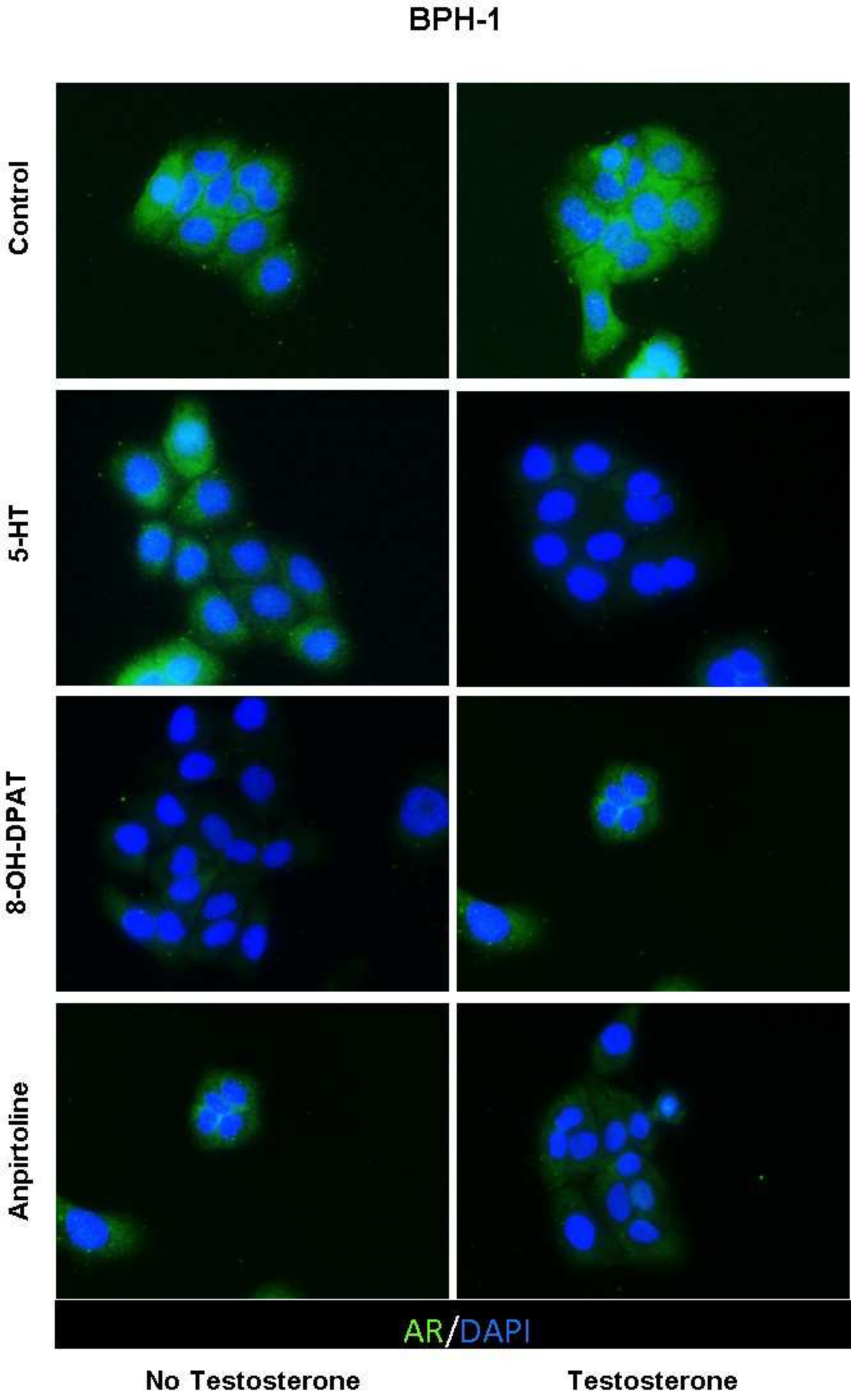

Supplementary Figure 2. Effect of 5-HT, *5-Htr1a* specific agonist and *5-Htr1b* specific agonist on AR expression in BPH-1 cells. Immunofluorescence analysis of AR expression after 5-HT, 8-OH-DPAT and Anpirtoline treatment in medium conditions without and with testosterone supplementation. AR, androgen receptor; 5-HT, serotonin.

Supplementary Figure 3

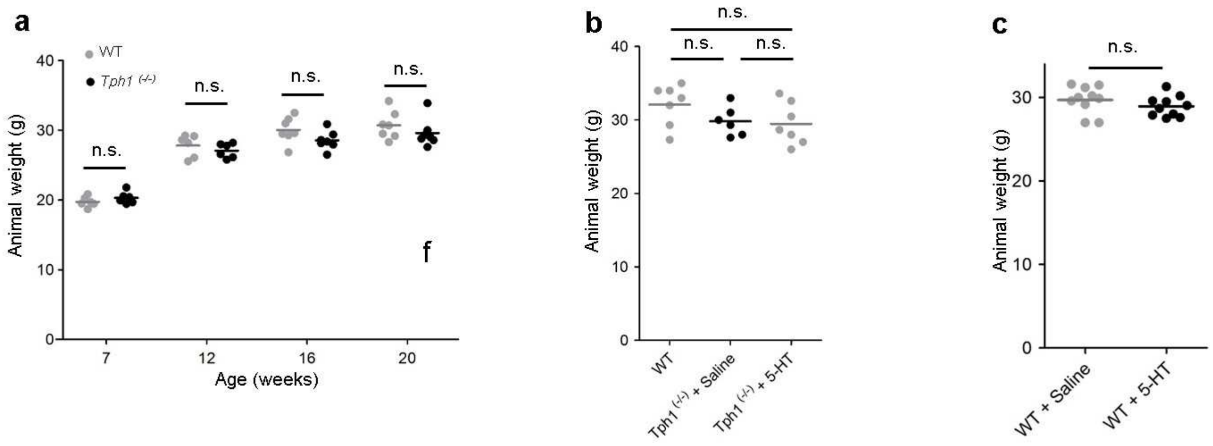

Supplementary Figure 3. Genetic deletion of Tph1 and 5-HT treatment does not affect animal weight. **(a)** Weight of wild-type and *Tph1*$^{-/-}$ mice at different ages. **(b)** Weight of wild-type, *Tph1*$^{-/-}$ treated with Saline and *Tph1*$^{-/-}$ mice treated with 5-HT during 10 consecutive days **(c)** Weight of wild-type treated with Saline and wild-type mice treated with 5-HT during 10 consecutive days. n.s., non-significant.
